# Monoamine Oxidase A Inhibits Lung Adenocarcinoma Cell Proliferation by Abrogating Aerobic Glycolysis

**DOI:** 10.3389/fonc.2021.645821

**Published:** 2021-03-08

**Authors:** Yumin Huang, Wei Zhao, Xiaoping Ouyang, Feng Wu, Yujian Tao, Minhua Shi

**Affiliations:** ^1^Department of Respiratory Medicine, The Second Affiliated Hospital of Soochow University, Suzhou, China; ^2^Department of Respiratory Medicine, The Affiliated Hospital of Yangzhou University, Yangzhou, China; ^3^School of Laboratory Medicine/Sichuan Provincial Engineering Laboratory for Prevention and Control Technology of Veterinary Drug Residue in Animal-Origin Food, Chengdu Medical College, Chengdu, China

**Keywords:** lung adenocarcinoma, monoamine oxidase A, aerobic glycolysis, hexokinase 2, cell prolferation

## Abstract

Lung adenocarcinoma (LUAD) accounts for ~30% of all lung cancers and is one of the causes of cancer-related death worldwide. As the role of monoamine oxidase A (MAOA) in LUAD remains unclear, in this study, we examine how MAOA affects LUAD cell proliferation. Analyses of both public data and our data reveal that the expression of MAOA is downregulated in LUAD compared with non-tumor tissue. In addition, the expression of MAOA in tumors correlates with clinicopathologic features, and the expression of MAOA serves as an independent biomarker in LUAD. In addition, the overexpression of MAOA inhibits LUAD cell proliferation by inducing G1 arrest *in vitro*. Further mechanistic studies show that MAOA abrogates aerobic glycolysis in LUAD cells by decreasing hexokinase 2 (HK2). Finally, the expression of HK2 shows a negative correlation with MAOA in LUAD, and high HK2 predicts poor clinical outcome. In conclusion, our findings indicate that MAOA functions as a tumor suppressor in LUAD. Our results indicate that the MAOA/HK2 axis could be potential targets in LUAD therapy.

## Introduction

Lung cancer is one of the most common cancers and the leading cause of cancer-related death globally (1). Lung adenocarcinoma (LUAD) accounts for more than 30% of all lung cancers and for about half of all non-small cell lung cancer (NSCLC) (2, 3). Alterations of gene expression and abnormal signal pathways affect the proliferation of lung cancer (4–7), which greatly limit the treatment options. The identification of molecules associated with LUAD tumor growth may not only shed light on the underlying biological mechanisms involved in the development or progression of the disease but also reveal potential novel targets for the LUAD therapy.

Monoamine oxidase A (MAOA) is an enzyme which breaks down adrenergic neurotransmitters, such as norepinephrine and dopamine, and is widely expressed in the liver, the digestive tract, the placenta, and the lung, among other tissues (8). MAOA is overexpressed in prostate tumors, and it promotes cancer cell proliferation, stemness, and tumorigenesis (9, 10). Clinical and *in vitro* data indicate that MAOA functions as a tumor suppressor in hepatocellular carcinoma (11), cholangiocarcinoma (12), pheochromocytoma (13), neuroblastoma (14), renal cell carcinoma (15), and oral and pharyngeal cancers (16). MAOA is overexpressed in NSCLC and stimulates the epithelial–mesenchymal transition in cancer cells (17, 18). An inhibitor of MAOA repressed paclitaxel-resistant NSCLC metastasis and growth (19). However, MAOA is expressed at a low level in LUAD compared to non-tumor tissues, and the overexpression of MAOA correlates with poor outcome for LUAD according to the samples from Gene Expression Profiling Interactive Analysis (GEPIA) (20), The Cancer Genome Atlas (TCGA), and The Genotype-Tissue Expression (GTEx). Thus, the expression and role of MAOA in LUAD needs further study.

Aerobic glycolysis provides abundant ATP, sufficient biomolecules (e.g., nucleotides, amino acids, and lipids), and signaling pathways that are regulated by glycolysis metabolites (21, 22). Reprogramed aerobic glycolysis promotes the proliferation of bladder cancer cells (23), breast cancer cells (24), and LUAD cells (25, 26). Hexokinase 2 (HK2) is the key rate-limiting enzyme in glycolysis and is overexpressed in NSCLC tumors and promotes cancer cell proliferation (27). Moreover, the repression of HK2 abrogates NSCLC tumor growth (28). These reports suggest that HK2 and its related molecules may be promising therapeutic targets in LUAD.

In the present study, we found a low level of MAOA in LUAD tumors and cell lines. The expression of MAOA correlated with LUAD clinicopathological factors and the clinical outcome. Aerobic glycolysis in LUAD cells was inhibited by MAOA in an HK2-dependent manner. Our results reveal the tumor suppressive role of MAOA in LUAD growth.

## Materials and Methods

### Specimen Collection

This study recruited 108 patients with lung cancer who received surgery in the Second Affiliated Hospital of Soochow University. The patients received no chemotherapy or radiotherapy before surgery. Samples from lung cancer tissues and non-tumor lung tissues (>5 cm from the tumor margin) were dissected, snapped frozen in liquid nitrogen after surgery, and stored at −80°C. Clinicopathological data, such as sex, age, smoking status, size of tumor, lymph nodal status, pathological differentiation, and clinical stage, were obtained at the time of surgery. The postoperative staging was determined according to the 7th Edition of the TNM classification (29). Pathological type was determined according to the classification by the WHO (30). This study was approved by the Research Ethics Committee of the Second Affiliated Hospital of Soochow University (ID: RD2019X011). Written informed consent was obtained from all patients.

### Immunohistochemistry (IHC) and Evaluation

Collected samples were fixed in 10% neutral buffered formalin and embedded in paraffin. Sections were cut at 5 μm and placed on slides coated with poly-L-lysine. Sections were deparaffinized in xylene and rehydrated in descending concentrations of alcohol. Antigen retrieval was achieved by boiling the sections in a 10 mm citric acid buffer (pH 6.0) for 25 min. Endogenous peroxidase activity was blocked by incubating sections with 3% hydrogen peroxide for 15 min. The sections were incubated with 10% normal goat serum for 15 min after two washes in a phosphate buffered saline (PBS). The sections were incubated with the rabbit anti-human MAOA polyclonal antibody (1:300, Bioworld, China) at 4°C overnight. Later, the sections were incubated with biotinylated goat anti-rabbit IgG antibody (1:100, Bioworld, China) at room temperature for 15 min, followed by incubating with streptavidin-biotinylated peroxidase at room temperature for another 15 min. The sections were washed in PBS, and the diaminobenzidine (DAB) solution was used to develop color. Color development was monitored under a bright-field microscope, and the reaction was stopped by dipping the sections in water. Hematoxylin was used for nuclear counterstaining.

The IHC staining was evaluated independently by two pathologists at the Second Affiliated Hospital of Soochow University in a double-blinded manner, as described previously (31). In cases of disagreement, a consensus was made through discussion. The stained tumor cells in four randomly selected high magnification fields were counted. The percentage of positively stained tumor cells was graded as 0, 1, 2, and 3, with 0 equals 0% or <5% tumor cells, 1 equals 5–25% tumor cells, 2 equals 25–50% tumor cells, and 3 equals >50% tumor cells. High expression of MAOA was defined as the IHC score ≥mean of total of tumors IHC score, whereas the low expression of MAOA was defined as the IHC score < mean IHC score of total tumors.

### Western Blotting Assay

Protein samples (20 μg) were electrophoresed on a 10% SDS-PAGE gel and transferred onto polyvinylidene difluoride membranes. The membranes were blocked and incubated overnight with antibodies against MAOA (Bioworld), HK2 (Bioworld), proliferating cell nuclear antigen (PCNA) (Santa Cruz, CA, USA), or β-actin (Bioworld, Nanjing, China). The membranes were then incubated with the corresponding horseradish peroxidase-conjugated secondary antibodies for 2 h at room temperature. Protein bands were detected using the Pierce SuperSignal West Pico Chemiluminescent Detection System (Thermo Fisher Scientific Inc., Rockford, IL, USA) and visualized in a G: BoxiChem Imager (Syngene, Cambridge, UK).

### Quantitative RT–PCR Analysis

Total RNAs were extracted using the TRIzol® Reagent (Invitrogen Inc., Carlsbad, CA, USA). Quantitative RT–PCR was used to examine the mRNA expression of MAOA using 2 μg total RNA, and β-actin was used as an internal control. The sequences of primers are as follows: MAOA forward 5′-TCCCGAGCTTCTAAAACCAA-3′ and reverse 5′-GGAGAATCAAGAGAAGGCGA-3′; *HK2* forward 5′-GGCTCTGGACAGGTGGTAAAGA-3′ and reverse 5′- CGGTAATGCACCACCTTGGTGT-3′; β-actin forward 5′-AGCGAGCATCCCCCAAAGTT-3′ and reverse 5′-GGGCACGAAGGCTCATCATT-3′. The qRT–PCR was performed on the ABI StepOne Sequence Detection System using the SYBR® Green (TaKaRa Biotechnology Co. Ltd., Dalian, China). The conditions used include 95°C for 10 min, followed by 40 cycles of 95°C for 5 s and 55°C for 31 s. The ΔCT (CT value of target gene—CT value of internal control) was used for the quantification of the transcripts.

### Cell Lines and Stable Cell Line Construction

The human NCSLC cell lines NCI-H1975 and A549 were obtained from the Cell Bank of Type Culture Collection of Chinese Academy of Sciences (Shanghai, China). The cells were cultured in RPMI 1640 Medium containing 10% FBS (Hyclone). The overexpression of MAOA in LUAD cell lines (NCI-H1975 and A549) was conducted using a lentivirus containing MAOA cDNA or control letivirus (constructed by GeneChem, Shanghai, China). Lentivirus infection was performed as previously reported (32).

### Cell Viability Assay

Cells (1.0 × 10^3^) were plated into each well of a 96-well-plate. A10 μl MTT solution was added to each well after 0, 24, 48, and 72 h of culture and incubated in the dark at 37°C for 4 h. After that, the medium was discarded. A 100 μl dimethyl sulfoxide (DMSO) was used to dissolve the formazan crystals. Light absorbance was read at 490 nm. The growth curves were determined from each experiment, which was repeated three times.

### Cell Cycle Analysis

The cell cycle was evaluated by flow cytometric analysis with propidium iodide (PI) for DNA staining. In brief, cells were harvested and washed in PBS. Later, the cells were fixed in cold 70% ethanol for 30 min at 4°C. Next, 50 μl of a 100 μg/ml stock of RNase (Sigma, St. Louis, MO, USA) was added, followed by the addition of 1 mg/ml PI (Sigma). The cells were incubated at 37°C for 30 min. The cells were then evaluated by a flow cytometer (BD FACS Calibur, BD Biosciences, San Jose, CA, USA).

### Aerobic Glycolysis and Detection of HK2 Activity

Agilent Seahorse XFe96 Analyzers (Beijing, China) were used to measure the extracellular acidification rate (ECAR) of cancer cells in a 96-well-plate followed the manufacturer's manual.

The glucose consumption and lactate production in stable MAOA overexpressing cells and the corresponding control cells were detected as follows. Cells were seeded into 35-mm dishes for 36 h. The supernatants of cell culture medium were collected by centrifugation at 800 rpm for 5 min. The Glucose Assay Kit (Sigma, Shanghai, China) and the Lactate Assay kit (BioVision, Milpitas, CA, USA) were used to determine the level of glucose and lactate, respectively. The PicoProbe™ Hexokinase Activity Assay Kit (BioVision, CA, USA) was used to detect HK2 activity, as previously reported (5).

### Colony Formation Assay

Non-small cell lung cancer cells (300) were plated into 6-well-plates. Cells were incubated for 14 days. The colonies were fixed using 100% methanol and stained with 0.5% crystal violet solution. Colonies with >50 cells were counted. Each experiment was performed in triplicate.

### Statistical Analysis

Results are expressed as mean ± SD. The significance of the difference between the experimental groups, expression correlation, and MAOA correlation with clinicopathologic features were evaluated by the χ^2^ analysis. The Kaplan–Meier curve and the log-rank test were used to analyze the survival of the patients. A value of *p* < 0.05 was considered significant for all analyses.

## Results

### MAOA Expression Is Decreased in LUAD

**Figure 1:**
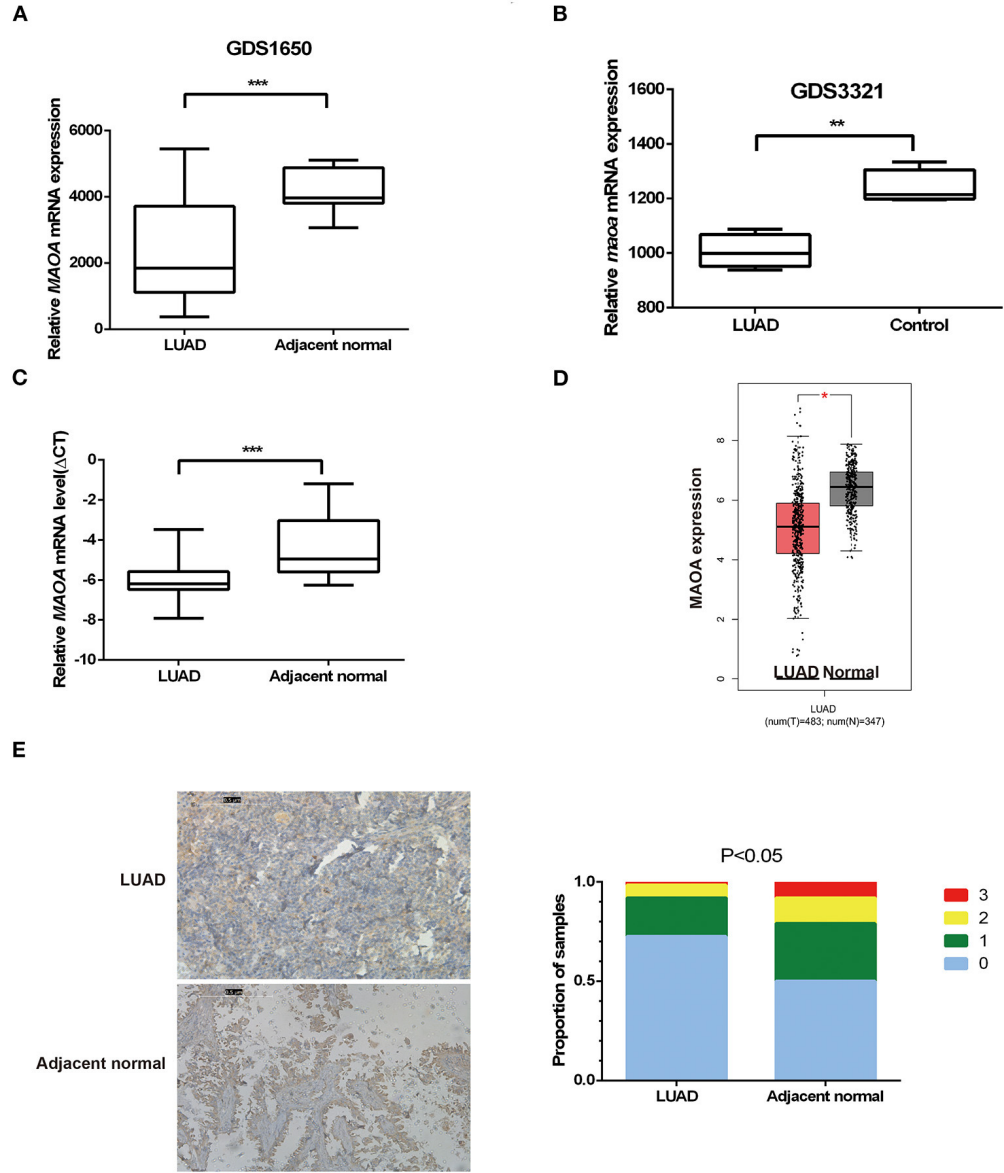
The expression of monoamine oxidase A (MAOA) in lung adenocarcinoma (LUAD). The expression of MAOA in gene expression omnibus (GEO) data **(A)**, 19 pairs of human LUAD specimens **(B)**, 15 pairs of mice induced LUAD, 108 human LUAD specimens **(C)**, and the gene expression profiling interactive analysis (GEPIA) database **(D)**. The immunohistochemistry (IHC) detection of MAOA in human LUAD specimens **(E)**. *p < 0.05, **p < 0.01, and ***p < 0.001, compared with non-tumor tissues.

The open database of cancer gene expression was examined for the expression of MAOA in gene expression omnibus (GEO) profile datasets (GDS1650 and GDS3321) (33, 34). The results showed that the MAOA mRNA level of LUAD tumor in both human (GDS1650) and mice (GDS3321) was significantly lower than the normal tissue (Figures 1A,B). We performed qRT-PCR to evaluate the MAOA mRNA expression in 108 paired LUAD samples and adjacent normal tissues. It was found that the MAOA mRNA level was significantly decreased in tumors compared with adjacent normal tissues (Figure 1C). Similar findings were observed in the GEPIA (20) database (Figure 1D). The IHC staining showed that the MAOA expression was reduced in LUAD specimens compared with non-tumor controls (Figure 1E), which was inconsistent with the mRNA results. These results suggest that both mRNA and the protein level of MAOA were decreased in LUAD, indicating that MAOA has a tumor suppressive role in LUAD cells.

### The Expression of MAOA Is Associated With Clinicopathologic Factors and the Survival of Patients With LUAD

**Figure 2:**
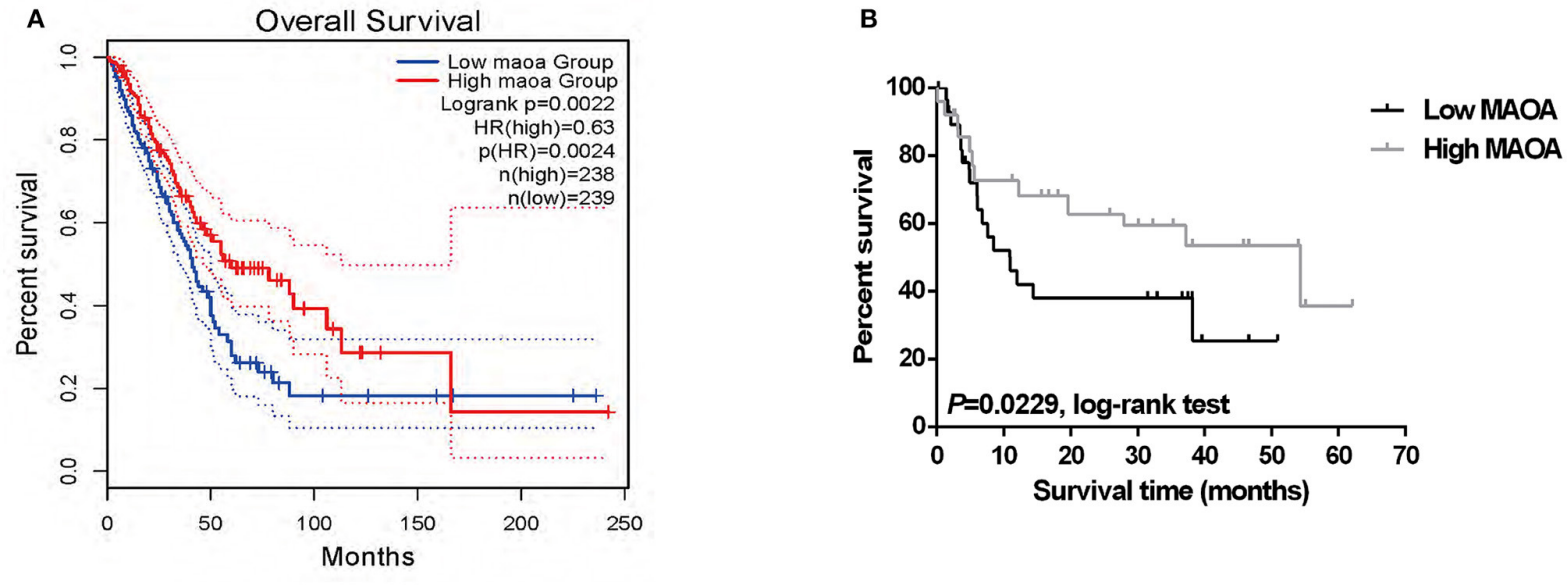
The expression of MAOA correlates with the survival of patients with LUAD. **(A)** Survival of the patients in two groups according to the median of MAOA mRNA level in GEPIA. **(B)** The survival of the high MAOA group and low MAOA group analyzed by the Kaplan–Meier analysis.

We investigated the correlation of the expression of MAOA with the overall survival in the GEPIA database of patients with LUAD. The results showed that high MAOA mRNA level indicated good clinical outcome compared to low MAOA mRNA level (Figure 2A). We classified 108 LUAD tumors according to MAOA expression and examined the low and high MAOA expression groups. The Kaplan–Meier survival analysis showed that higher MAOA predicted better clinical benefit (Figure 2B). Moreover, the expression of MAOA was significantly correlated with the lymph node metastasis status, tumor stage, gender, and smoking status (Table 1). Finally, multivariate analysis uncovered that the expression of MAOA is an independent prognostic biomarker for LUAD (Table 2). These results demonstrate that reduced expression of MAOA could be a promising prognostic biomarker for LUAD.

**Table 1:** MAOA IHC scores and clinicopathological factors in LUAD patients.

**Characteristic**	**Number of patients (%)**	**MAOA IHC scores**		
		**Low**	**High**	***P*-value**
All patients	108	71	37	
Gender				0.042^*^
Male	85 (78.7)	60	25	
Female	23 (21.3)	11	12	
Age				0.505
<65	66 (61.1)	45	21	
≥65	42 (38.9)	26	16	
Size of tumor				0.438
≤ 3 cm	30 (27.8)	18	12	
>3 cm	78 (72.2)	53	25	
Smoking status				0.009^*^
No Smoking	40 (37.0)	20	20	
Smoking	68 (63.0)	51	17	
Lymph node metastasis (pN)				0.003^*^
N0	65 (60.2)	50	15	
N1+N2+N3	43 (39.8)	21	22	
p-TNM stages				0.013^*^
I	51 (47.3)	40	11	
II	40 (37.0)	22	18	
III	16 (14.8)	8	8	
IV	1 (0.9)	1	0	

* *Statistically significant difference (P < 0.05)*.

**Table 2:** Multivariate analyses of MAOA expression and other clinical prognostic factors in 108 patients with LUAD.

**Factors**	**HR (95%CI)**	***P***
Age (>65/ ≤ 65 Years)	0.585 (0.330–1.039)	0.068
Gender (Femle/Male)	0.689 (0.351–1.353)	0.279
Smoking status (Yes/No)	2.452 (0.414–4.537)	0.743
Size of tumor (>3 cm/ ≤ 3 cm)	0.735 (0.414–1.303)	0.291
N stage (N_1+2+3_/N_0_)	1.734 (1.383–2.665)	0.023^*^
Stage (I and II vs. III and IV)	1.932 (1.312–2.675)	0.005^*^
MAOA expression (high/low)	2.125 (1.644–2.760)	0.008^*^

HR, hazard ration; 95% CI, 95% confidence interval.

* *Statistically significant difference (P < 0.05)*.

### MAOA Inhibits LUAD Cell Colony Formation and Proliferation

**Figure 3:**
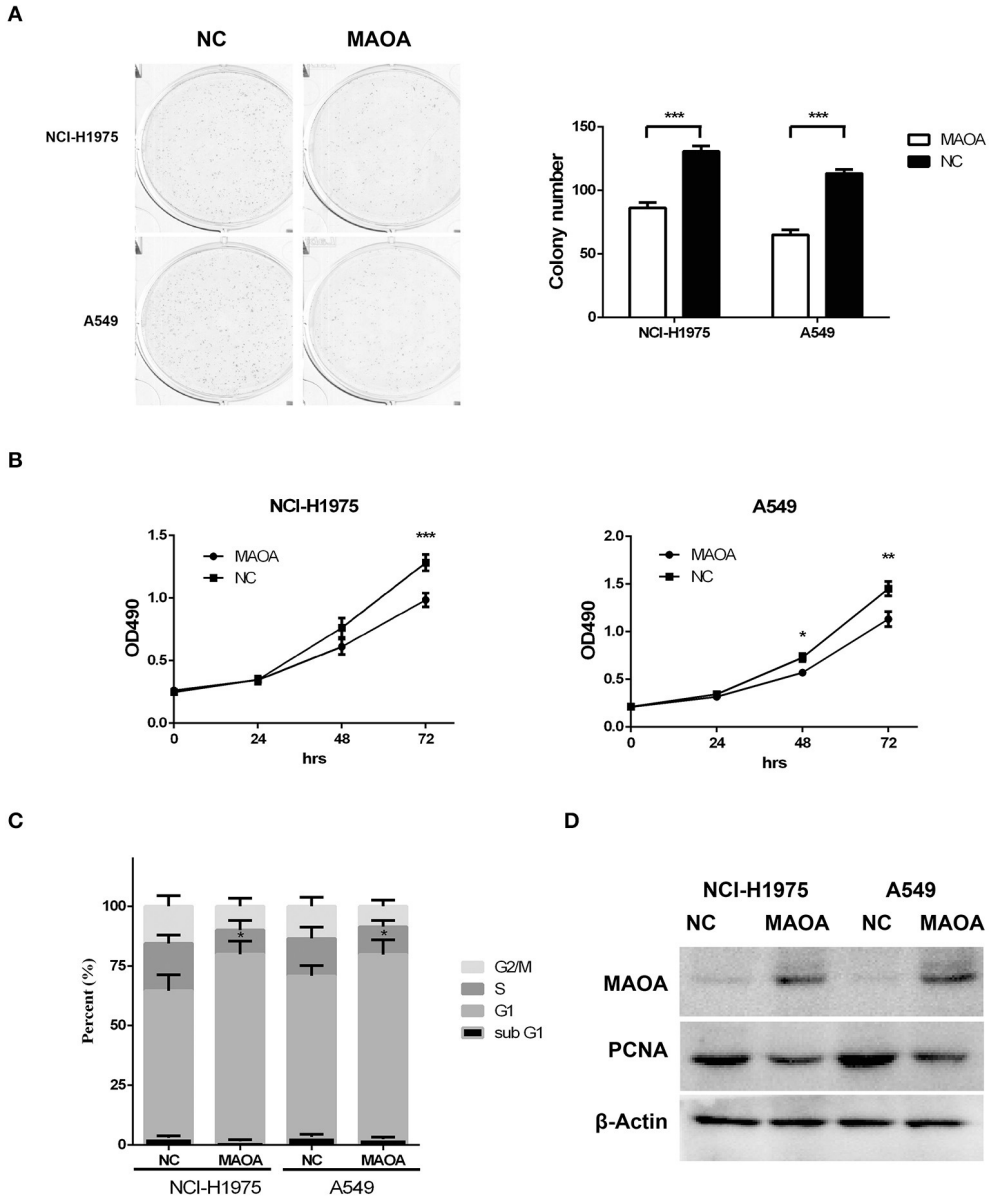
Overexpressed MAOA affects LUAD cancer cell proliferation and growth *in vitro*. Colony formation **(A)** and MTT **(B)** assays performed to evaluate the effects of MAOA on LUAD cells (NCI-H1975 and A549). Flow cytometry **(C)** and Western blot **(D)** performed to analyze LUAD cell cycle and the G1/S cell cycle-related protein proliferating cell nuclear antigen (PCNA), respectively. Data are presented as mean + SD (*n* = 3). *p < 0.05, **p < 0.01 and ***p < 0.001, compared to the control group.

To further examine the biological effect of MAOA, we overexpressed MAOA by a lentivirus in NCI-H1975 and A549 cells (Figure 3D). Colony formation assay revealed that the number of colonies of LUAD cells was significantly decreased by the overexpression of MAOA (Figure 3A). MAOA also significantly reduced cell proliferation at 72 h after the infection of the lentivirus (Figure 3B). Flow cytometry revealed that MAOA induced G1 arrest in LUAD cells (Figure 3C), suggesting that cell cycle arrest may be the major cause of MAOA in inhibiting cell proliferation and growth. Moreover, the cell cycle molecule PCNA was decreased in LUAD cells with the overexpression of MAOA (Figure 3D).

### MAOA Abrogates Aerobic Glycolysis in LUAD Cells

**Figure 4:**
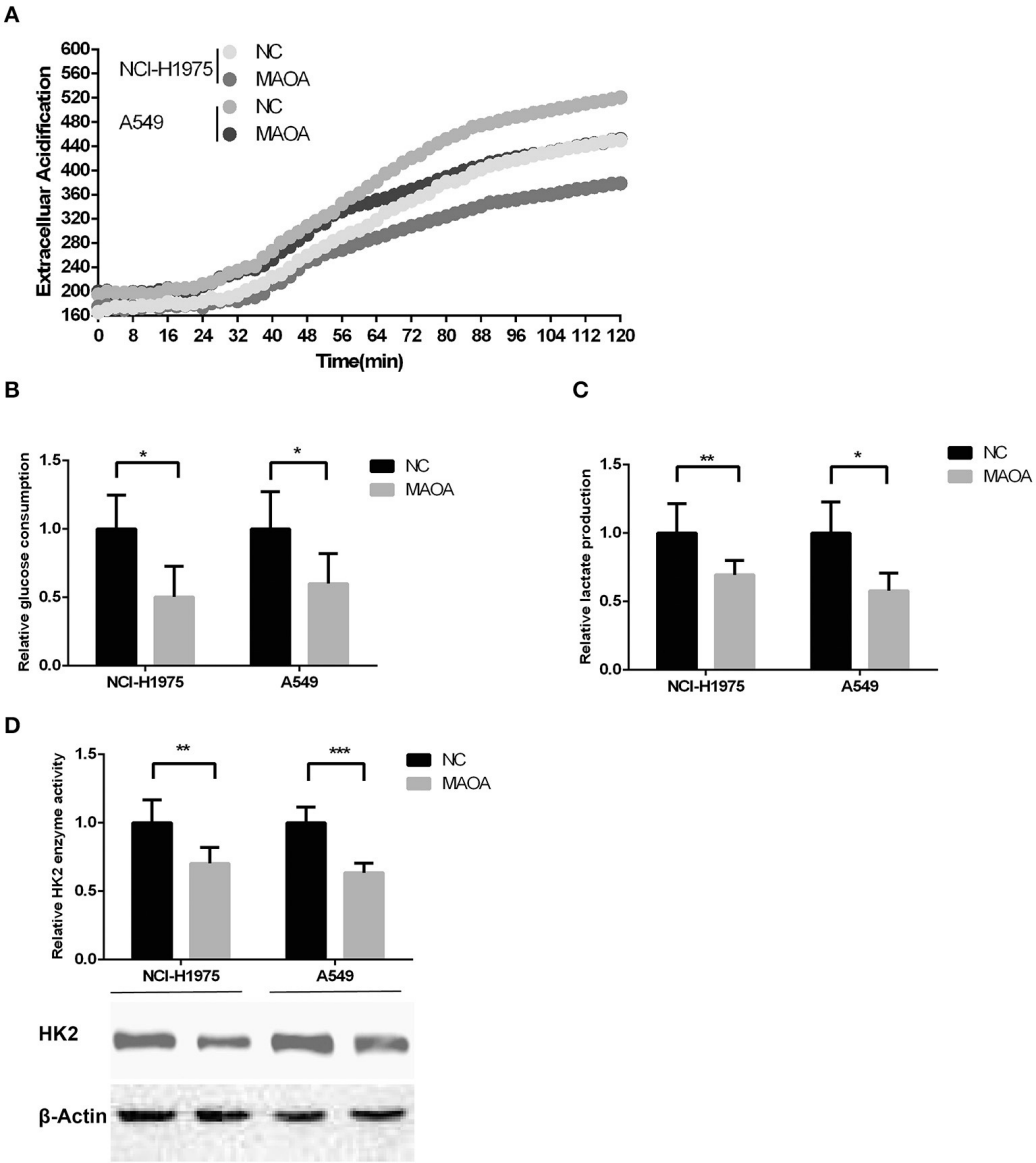
Aerobic glycolysis in MAOA-overexpressing LUAD cells. **(A)** Extracellular acidification rate [ECAR, **(B)** glucose consumption, and **(C)** lactate production were analyzed in MAOA-overexpressing and control LUAD cells. **(D)** The enzymatic activity and protein level of hexokinase 2 (HK2)] were detected by hexokinase activity assay and Western blot, respectively. Data are presented as mean + SD (*n* = 3). *p < 0.05, **p < 0.01, and ***p < 0.001, compared to the control group.

As aerobic glycolysis plays a critical role in LUAD growth and proliferation, we examined whether MAOA affects aerobic glycolysis in LUAD cells by detecting the ECAR. As shown in Figure 4A, the ECAR was reduced by the overexpression of MAOA in NCI-H1975 and A549 cells at around 60 min, and the difference was increased at 120 min. Consistent with these findings, glucose consumption and lactate production (Figures 4B,C) were significantly abrogated in LUAD cells with the overexpression of MAOA. Furthermore, the expression and enzymatic activity of HK2, a key rate-limiting enzyme in aerobic glycolysis, was reduced by the overexpression of MAOA (Figure 4D). These results suggest that MAOA regulates LUAD cell growth and proliferation by reducing aerobic glycolysis.

### HK2 Expression Negatively Correlates With MAOA Expression and Poor Clinical Outcome in LUAD

**Figure 5:**
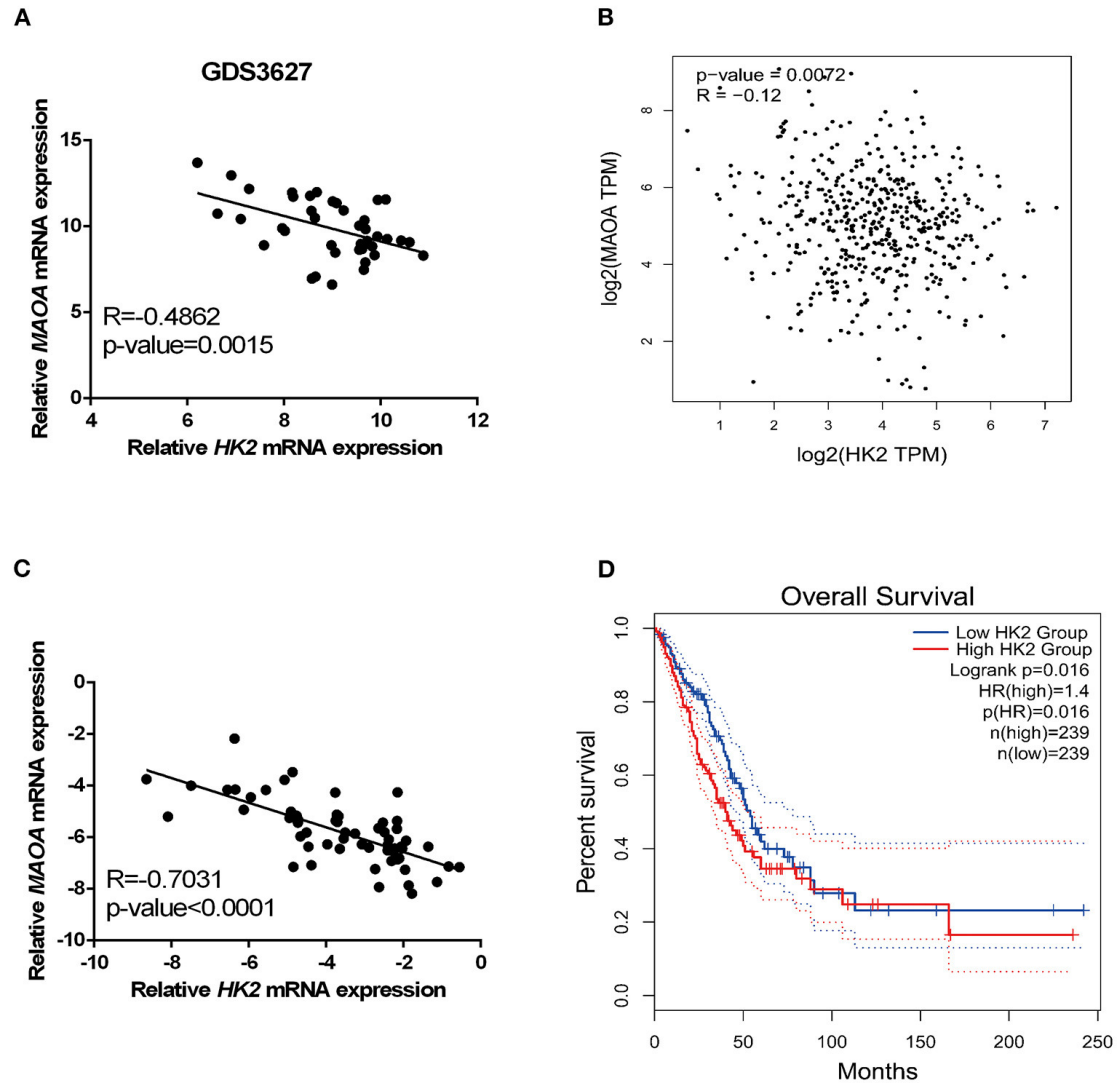
HK2 correlates negatively with MAOA in LUAD. The correlation between HK2 and MAOA was analyzed in the GEO dataset **(A)**, GEPIA **(B)**, and our collected LUAD tumors **(C)**. The survival rate of patients with LUAD was analyzed in GEPIA according to the expression of HK2 **(D)**.

Analyses of the GEO profile dataset (GDS3627) and GEPIA database revealed a negative correlation of the mRNA level between HK2 and MAOA in clinical specimens (Figures 5A,B). In our collected LUAD tissue samples, HK2 and MAOA have negatively correlated the expression in LUAD tumors (Figure 5C). Investigation of the GEPIA database also showed that LUAD cases with high expression of HK2 had a poor survival rate (Figure 5D). These results indicate that the MAOA/HK2 axis plays an important role in the progression of LUAD.

## Discussion

Lung adenocarcinoma accounts for ~30% of lung cancers. LUAD is the most common cancer and the leading cause of cancer-related death globally (35, 36). Thus, the identification of reliable predictive biomarkers and potential therapeutic targets for LUAD is urgently needed. In the present study, we demonstrated that the expression of MAOA was decreased in LUAD and correlated with the overall survival of patients. Additionally, we showed that MAOA abrogates cancer cell growth and serves as an independent biomarker for LUAD.

Monoamine oxidase A was reported to be an oncogene in NSCLC (17, 37). Tang's group found positive expression of MAOA in 9 out of 12 LUAD tumors by IHC (18). Recently, we reported that MAOA plays a critical role in NSLC migration and HPV-16 E7 induced-HIF-1α protein accumulation in NSCLC cells (17). Another group found that a potential inhibitor of MAOA, G11, increases the sensitivity of chemotherapy drug and metastasis of NSCLC cells (19). However, we found that MAOA was downregulated in LUAD in an open public database and 108 clinical specimens. Both the database and our results show that high expression of MAOA in LUAD correlated with better clinical outcome of patients with LUAD. Furthermore, MAOA works as an independent biomarker for LUAD prediction. The contradictory results on findings of this study and others might have arrived due to differences in the number of clinical specimens or detection methods.

Our results showed that the expression of MAOA correlates with the smoking status. Previous reports showed that MAOA is inhibited by tobacco smoke (38, 39), and smoking is the major cause of LUAD (40). These findings suggest that smoking may cause the downregulation of MAOA in LUAD.

In this study, overexpressed MAOA reduced LUAD cell growth and proliferation by inducing G1 cell cycle arrest. Emerging evidence indicates that enhanced aerobic glycolysis promotes transcription in the G1 phase and furnishes more ATP, which is necessary for the G1/S transition (41). Consistent with these findings, we found that the aerobic glycolysis of cancer cells was abrogated by MAOA. Moreover, MAOA reduced the protein level and enzyme activity of HK2, a key rate-limiting enzyme in glycolysis. These data suggest that MAOA inhibits LUAD cell growth and proliferation by abrogating HK2-dependent aerobic glycolysis.

In conclusion, the expression of MAOA showed a negative correlation with HK2 in LUAD tumors, and overexpressed MAOA reduced the expression of HK2 and the enzymatic activity. MAOA could be a potential therapeutic target for LUAD treatment.

## Data Availability Statement

The original contributions generated for the study are included in the article/supplementary material, further inquiries can be directed to the corresponding author/s.

## Ethics Statement

The studies involving human participants were reviewed and approved by The Research Ethics Committee of the Second Affiliated Hospital of Soochow University. The patients/participants provided their written informed consent to participate in this study.

## Author Contributions

MS: conceptualization. YH, WZ, and XO: methodology. YH, WZ, FW, and YT: validation. WZ: formal analysis. MS, YH, and WZ: investigation and writing–original draft preparation. YH: resources. MS and YH: data and supervision. YH, WZ, and XO: writing, reviewing, and editing. MS and WZ: funding acquisition. All authors have read and agreed to the final version of the manuscript.

## Conflict of Interest

The authors declare that the research was conducted in the absence of any commercial or financial relationships that could be construed as a potential conflict of interest.
